# *Inquérito Telefônico de Fatores de Risco para Doenças Crônicas Não Transmissíveis em Tempos de Pandemia* (Covitel): aspectos metodológicos 

**DOI:** 10.1590/0102-311XPT248922

**Published:** 2023-09-25

**Authors:** Pedro Curi Hallal, Angel Caroline Chirivino Antunes da Rocha, Luciana Monteiro Vasconcelos Sardinha, Aluísio J. D. Barros, Fernando C. Wehrmeister

**Affiliations:** 1 Programa de Pós-graduação em Epidemiologia, Universidade Federal de Pelotas, Pelotas, Brasil.; 2 Department of Kinesiology and Community Health, University of Illinois Urbana-Champaign, Urbana, U.S.A.; 3 Vital Strategies, São Paulo, Brasil.

**Keywords:** Vigilância Sanitária, Doenças Crônicas, Adultos, Inquéritos Epidemiológicos, Health Surveillance, Chronic Diseases, Adults, Health Surveys, Vigilancia Sanitaria, Enfermedades Crónicas, Adultos, Encuestas Epidemiológicas

## Abstract

Este artigo descreve a metodologia utilizada na realização do *Inquérito Telefônico de Fatores de Risco para Doenças Crônicas Não Transmissíveis em Tempos de Pandemia* (Covitel), desenvolvido no Brasil em 2022. O Covitel é um inquérito de base populacional, com representatividade para o Brasil e suas cinco macrorregiões: Centro-oeste, Nordeste, Norte, Sudeste e Sul. O inquérito apresenta informações sobre o impacto dos principais fatores de risco para as doenças crônicas não transmissíveis (DCNT) na população adulta, com 18 anos ou mais, residente em domicílios servidos por linhas telefônicas fixas e móveis. O estudo tem por objetivo colaborar para o desenvolvimento e acompanhamento de políticas públicas voltadas para a promoção da saúde para a população, bem como obter resultados que visem contribuir para o conhecimento sobre a influência da COVID-19 nos fatores de risco para as DCNT no país. Foram avaliados 9 mil indivíduos e coletadas informações sobre alimentação, atividade física, saúde mental, estado de saúde, hipertensão arterial, diabetes e depressão, além do consumo de álcool e tabaco, comparando os momentos pré-pandemia e o primeiro trimestre de 2022. Além disso, o estudo coletou informações acerca do esquema vacinal da população e da infecção por COVID-19.

## Abstract (en)

This study describes the methodology of the *Telephone Survey of Risk Factors for Chronic Noncommunicable Diseases During the Pandemic* (Covitel), conducted in Brazil in 2022. Covitel is a population-based survey representing Brazil and its five macroregions (Central-West, Northeast, North, Southeast, and South) and providing information on the impact of the main risk factors for chronic noncommunicable diseases (NCDs) on the adult population aged 18 years or above who live in households served by fixed and mobile telephone lines. This study aims to contribute to the development and monitoring of public policies to promote the population’s health and obtain results to contribute to the knowledge of the influence of COVID-19 on risk factors for NCDs in the country. We evaluated 9,000 individuals and collected information on their diet, physical activity, mental health, health status, hypertension, diabetes, depression, and alcohol and tobacco consumption, comparing the pre-pandemic moments and the first quarter of 2022. We also collected information about the population’s vaccination schedule and COVID-19 infection history.

## Resumen

Este artículo describe la metodología empleada para realizar la *Encuesta Telefónica de Factores de Riesgo para Enfermedades Crónicas No Transmisibles en Tiempos de Pandemia* (Covitel), realizada en Brasil en el 2022. Covitel es una encuesta de base poblacional, representativa de Brasil y sus cinco macrorregiones: Centro-Oeste, Nordeste, Norte, Sudeste y Sur, y brinda información sobre el impacto de los principales factores de riesgo para enfermedades crónicas no transmisibles (ECNT) en la población adulta, de 18 años o más, que vive en hogares con servicio de telefonía fija y móvil. El estudio tiene como objetivo contribuir al desarrollo y seguimiento de políticas públicas dirigidas a la promoción de la salud de la población, así como obtener resultados que tengan como objetivo contribuir al conocimiento sobre la influencia de la COVID-19 en los factores de riesgo para las ECNT en el país. Se evaluó a 9.000 individuos y se recopiló información sobre alimentación, actividad física, salud mental, estado de salud, hipertensión arterial, diabetes y depresión, además del consumo de alcohol y tabaco, comparando los momentos previos a la pandemia con el primer trimestre de 2022. Además, el estudio recopiló información sobre el calendario de vacunación de la población y la infección por COVID-19.

## Introdução

As doenças crônicas não transmissíveis (DCNT) representam 71% de todas as mortes no mundo ^1^. Mais de três quartos dos óbitos globais por DCNT ocorrem em países de renda média ou baixa ^2^. No Brasil, essas doenças respondem por 72% das causas de morte da população ^1^^,^^3^. As DCNT têm como características longa duração e progressão lenta e estão fortemente relacionadas a fatores de risco comuns e modificáveis. Entre eles, destacam-se inatividade física, alimentação inadequada, excesso de peso, tabagismo, poluição do ar e consumo abusivo de álcool ^4^^,^^5^. Recentemente, estudos evidenciam um aumento dos principais fatores de risco durante o período pandêmico da COVID-19, nacional e internacionalmente.

Diante do desafio global de prevenção e controle das DCNT, o monitoramento dos fatores de risco representa uma importante estratégia para contenção da progressão de doenças crônicas. Tendo em vista o momento pandêmico enfrentado, se fez necessária a realização emergencial de coletas de dados que forneçam evidências no contexto brasileiro. O *Inquérito Telefônico de Fatores de Risco para Doenças Crônicas Não Transmissíveis em Tempos de Pandemia* (Covitel) surgiu da importância de conhecer a influência da COVID-19 sobre os fatores de risco para as DCNT no Brasil.

Trata-se de um inquérito de representatividade nacional e das cinco macrorregiões do país, que apresenta informações robustas e atualizadas sobre o impacto dos principais fatores de risco para DCNT na população adulta, com 18 anos ou mais. Cientes da importância desses dados para a vigilância em saúde e para o planejamento de políticas públicas nessa área, temos como objetivo apresentar os aspectos metodológicos do Covitel.

## Aspectos metodológicos

### Amostragem

Como plano amostral, adotou-se a estratégia de obtenção de amostras probabilísticas da população brasileira, tendo como domínios as macrorregiões estabelecidas pela divisão do Instituto Brasileiro de Geografia e Estatística (IBGE): Norte, Nordeste, Centro-oeste, Sudeste e Sul. O estudo teve como população-alvo indivíduos com idade superior ou igual a 18 anos que tivessem linhas telefônicas fixa ou móvel.

A amostra foi composta por 1.800 indivíduos de cada região brasileira (900 para telefonia fixa e outros 900 para telefonia móvel), totalizando 9 mil pessoas. Esse número foi escolhido para se obter estimativas de prevalência confiáveis para qualquer fator de risco na população estudada, com nível de 95% de confiança e margem de erro de cerca de três pontos percentuais para cada grande região.

A seleção de linhas telefônicas ocorreu por meio do método de discagem aleatória de dígitos (RDD, do inglês *random digit dialing*). Para garantir a representatividade de cada uma das cinco regiões, foi considerada a proporção dos códigos de discagem direta à distância (DDD) de cada região. Esse procedimento era realizado da seguinte forma: (1) em uma planilha de Excel (https://products.office.com/), eram identificados e listados os números de DDD de cada uma das regiões; (2) foi gerado aleatoriamente o primeiro dígito do telefone, limitando-se aos números entre 2 e 5 para telefonia fixa e 6 a 9 para telefonia móvel (o algarismo 1 representa serviços de utilidade pública); (3) os demais, com algarismos de 0 a 9, eram posteriormente gerados de forma aleatória. As duplicatas foram removidas e os números restantes eram selecionados por sorteio e enviados para validação mecânica, por meio do uso de uma discadora eletrônica. Foram considerados números inválidos os telefones inexistentes ou empresariais, representando um total de 14,7% das ligações totais (sendo 75,2% de tentativas realizadas em linhas fixas e 24,8% móveis).

A segunda etapa da amostragem consistiu na seleção do indivíduo entrevistado pelo sistema e na obtenção do consentimento para participar da entrevista. Foram consideradas inelegíveis as linhas fora de serviço, que correspondessem a empresas ou que não existissem mais, além daquelas que não tivessem respondido a seis chamadas feitas em dias e horários variados, incluindo sábados e domingos e período noturno. As ligações foram realizadas entre 09:00 e 21:45 em dias de semana e entre 10:00 e 16:00 nos fins de semana. Quanto à distribuição das ligações de acordo com os turnos diários, elas representaram 15% das ligações realizadas pela manhã, 53% pela tarde, 22% no período da noite e 11% aos fins de semana. Para as linhas de telefonia móvel, foi entrevistado o responsável pela linha, caso tivesse idade maior ou igual a 18 anos. Para as linhas de telefonia fixa, foram ordenados todos os moradores com 18 anos ou mais em ordem crescente de idade e, então, sorteado um deles para compor a amostra do estudo. Caso o morador sorteado não estivesse presente no ato do sorteio, era agendado dia e horário para nova ligação e aplicação do questionário.

### Coleta de dados

As entrevistas telefônicas foram feitas entre os meses de janeiro e março de 2022 por uma empresa especializada. Para realização do procedimento de coleta de dados, foi necessária uma equipe técnica, composta por operadores, monitores, auxiliares, supervisores e coordenador de campo. Toda a equipe foi treinada e padronizada para a coleta. Além disso, todos os profissionais se comprometeram a manter sigilo sobre as informações coletadas.

O coordenador de campo era responsável pela seleção da equipe, bem como pelo acompanhamento da coleta de dados, realizando agendamentos de entrevistas e controle de qualidade de todos os procedimentos da equipe. Os monitores eram responsáveis por auxiliar os supervisores no controle de qualidade das entrevistas e na avaliação dos operadores por meio de auditorias. Já os supervisores eram responsáveis por preparar os procedimentos de coleta de dados, distribuindo planilhas, revisando e checando as entrevistas e os conteúdos gravados, solucionando possíveis problemas ou falhas. Os auxiliares eram responsáveis por assessorar os operadores, participando da coleta de dados in loco, auxiliando e sanando dúvidas no processo da coleta sempre que necessário. Por fim, os operadores eram encarregados da condução das entrevistas, realizando as ligações telefônicas, fazendo a abordagem inicial e aplicando o questionário no ato.

Para garantir a qualidade das entrevistas, foram feitas gravações, auditorias e checagem em 10% da amostra, escolhidas de forma aleatória. As auditorias permitiam monitorar a aplicação correta do questionário e a abordagem do entrevistador. Já a checagem teve por objetivos averiguar a clareza na realização das perguntas e sanar possíveis dúvidas sobre o projeto. Dessa forma, o supervisor acompanhava a tela do operador durante a aplicação do questionário, sem que ele soubesse.

### Instrumento para coleta de dados

O questionário do Covitel foi estruturado de maneira a viabilizar a condução das entrevistas com o uso de computadores, em que as respostas foram imediatamente registradas em meio eletrônico e estão disponíveis em domínio digital no *link*: http://observatoriodaaps.com.br/covitel/. Além disso, o questionário utilizado para a realização das entrevistas apresentou o máximo de semelhança com outros instrumentos utilizados na área, a fim de proporcionar comparabilidade entre estudos.

De modo geral, o questionário era composto por 45 questões gerais, englobando seis blocos de perguntas relacionados a: (1) características sociodemográficas; (2) características sobre frequência de consumo de alimentos e obesidade; (3) atividade física (lazer, deslocamento, no domicílio e no trabalho) e tempo de tela; (4) frequência de consumo de cigarros e bebidas alcoólicas; (5) informações sobre morbidade e autopercepção do estado de saúde; e (6) informações sobre infecção por COVID-19 e vacinação contra a doença.

Com o objetivo de comparar diferentes momentos temporais, as questões foram estruturadas de modo a abordar os períodos atual (últimos três meses) e pré-pandemia (três meses anteriores ao início da pandemia). Dessa forma, a respeito das características sobre padrão de alimentação, os indivíduos foram questionados sobre a frequência do consumo de verduras ou legumes, frutas, refrigerantes ou sucos artificiais e bebidas alcoólicas. Quanto às características sobre padrão de atividade física, eram abordadas questões referentes à prática de atividade física nos domínios de lazer, deslocamento, ocupacional e doméstico, além do uso de telas. Por fim, o bloco sobre estado de saúde englobava perguntas sobre percepção do estado de saúde (bom ou muito bom) e diagnósticos autorreferidos de hipertensão, diabetes e depressão.

### Operacionalização dos indicadores

A partir dessas questões, foram desenvolvidos indicadores de interesse para elaboração do relatório final do Covitel. Eles estão disponíveis no endereço eletrônico citado anteriormente (http://observatoriodaaps.com.br/covitel/). As informações utilizadas para a construção de indicadores de interesse estão descritas no Quadro 1.

**Quadro 1:** Informações disponíveis a partir do questionário de indicadores de interesse elaborado pelo *Inquérito Telefônico de Fatores de Risco para Doenças Crônicas Não Transmissíveis em Tempos de Pandemia* (Covitel). Brasil, 2022.

BLOCO DE QUESTÕES	PERÍODO DE INFORMAÇÃO EM RELAÇÃO À PANDEMIA		QUESTÕES DE INTERESSE
	ANTES	DURANTE	
Características sociodemográficas	-	-	Idade; sexo; escolaridade; cor da pele autorreferida; presença de plano de saúde; estado civil
Frequência alimentar, excesso de peso e obesidade	Sim	Sim	Consumo de legumes e verduras; consumo de frutas; consumo de refrigerante e sucos artificiais; peso e estatura autorreferidos
Atividade física e tempo de tela	Sim	Sim	Atividade física nos domínios de lazer, deslocamento, doméstico e ocupacional; uso de dispositivos eletrônicos
Consumo de cigarros e bebidas alcoólicas	Sim	Sim	Cigarro convencional; experimentação de narguilé; experimentação de cigarro eletrônico; consumo de álcool; consumo excessivo de álcool
Morbidade autorreferida e percepção de saúde	Sim	Sim	Diagnóstico médico de hipertensão arterial; diagnóstico médico de diabetes; diagnóstico médico de depressão; percepção do estado de saúde
Infecção e vacinação contra COVID-19	Não	Sim	Suspeita de COVID-19 sem confirmação diagnóstica; COVID-19 confirmada; vacinação contra COVID-19

### Operacionalização de características socioeconômicas e demográficas

Para o relatório final do Covitel, foram usadas variáveis sociodemográficas para descrever e caracterizar a amostra e os indicadores de interesse. As variáveis sociodemográficas estão descritas na Tabela 1 e foram operacionalizadas da seguinte forma: macrorregião do país (Norte, Nordeste, Centro-oeste, Sul e Sudeste), sexo do entrevistado (masculino e feminino), faixa etária (18-24 anos, 25-34 anos, 35-44 anos, 45-54 anos, 55-64 anos e 65 anos ou mais), cor da pele autorreferida (branca, parda, preta e outras), escolaridade (0-8 anos, 9-11 anos e 12 anos ou mais), plano de saúde (sempre teve, apenas antes da pandemia, apenas no momento da entrevista e nunca teve), estado civil (vive com ou sem companheiro) e trabalho (sempre teve, apenas antes da pandemia, apenas no momento da entrevista e nunca teve).

**Tabela 1:** Dados de caracterização da amostra, de acordo com variáveis demográficas e socioeconômicas, geral e por tipos de telefone. *Inquérito Telefônico de Fatores de Risco para Doenças Crônicas Não Transmissíveis em Tempos de Pandemia* (Covitel), Brasil, 2022.

Variáveis	Amostra geral		Amostra por telefone fixo		Amostra por celular	
	n	%	n	%	n	%
Sexo						
Masculino	3.768	41,8	1.572	34,9	2.196	48,8
Feminino	5.236	58,2	2.929	65,1	2.307	51,2
Faixa etária (anos)						
18-24	790	8,8	466	10,3	324	7,2
25-34	1.440	15,9	518	11,5	922	20,5
35-44	2.216	24,6	885	19,7	1.331	29,5
45-54	1.792	19,9	742	16,5	1.050	23,3
55-64	1.113	12,4	591	13,1	522	11,6
65 ou mais	1.653	18,4	1.299	28,9	354	7,9
Raça/Cor						
Branca	3.873	43,0	1.994	44,3	1.879	41,7
Preta/Parda	4.600	51,1	1.616	49,9	2.356	52,3
Outros	531	5,9	1.663	5,8	268	6,0
Escolaridade (anos de estudo)						
0-8	2.250	25,1	1.203	26,8	1.047	23,3
9-11	3.172	35,4	1.616	36,1	1.556	34,7
12 ou mais	3.548	39,5	1.663	37,1	1.885	42,0
Plano de saúde						
Sempre teve	3.809	42,6	2.033	45,5	1.776	39,7
Apenas antes	585	6,5	314	7,0	271	6,1
Apenas atualmente	428	4,8	194	4,4	234	5,2
Nunca teve	4.111	46,1	1.922	43,1	2.189	49,0
Trabalho						
Sempre trabalhou	4.755	52,9	1.783	39,7	2.972	66,1
Apenas antes	907	10,1	455	10,1	452	10,0
Apenas atualmente	507	5,6	243	5,4	264	5,9
Nunca trabalhou	2.821	31,4	2.013	44,8	808	18,0

### Inferências para a população maior de 18 anos, por macrorregião e país

A amostra foi dividida em estratos, considerando região geográfica (Nordeste, Norte, Sudeste, Sul e Centro-oeste), sexo (masculino e feminino), idade (18-34; 35-49 e 50 anos completos ou mais) e escolaridade (0-11 e 12 anos de escolaridade completos ou mais). Para calcular o peso amostral, visando representar a população brasileira e dessas regiões, foram obtidos dados do Sistema IBGE de Recuperação Automática (Sidra; tabela 3450, amostra do *Censo Demográfico* de 2010) ^6^. Como as categorias da tabela não são exatamente as mesmas do estudo, adaptações foram necessárias:

I) Idade - o IBGE trabalha com uma categoria de 15 a 19 anos. Para chegar ao número da população de 18 e 19 anos, foi feita uma estimativa simples de que a faixa de 18-19 anos corresponde a 2/5 da população de 15-19 anos. Visto que não existe grande variação no número de nascimentos ano a ano, a aproximação parece ser adequada. Em seguida, os grupos do IBGE foram somados de forma a produzir os *n* para os grupos do estudo: 18-34 anos, 35-49 anos e 50+ anos.

II) Escolaridade - o IBGE apresenta os dados por etapas de ensino. Todos os grupos abaixo do Ensino Médio foram inseridos na categoria de 0 a 11 anos de estudo; e o restante na categoria de 12 anos ou mais. Existe também um grupo indeterminado que foi somado ao grupo de 0-11 anos de estudo.

Assim, estimamos a população em 60 estratos, considerando a macrorregião geográfica (5) X sexo (2) X idade (3) X escolaridade (2). Não houve necessidade de serem utilizadas projeções de população, visto que nos interessava apenas a proporção do nosso tamanho amostral em relação à população.

Como a amostra do Covitel elenca indivíduos com base no DDD de registro da linha telefônica, estratégia equivalente a uma amostragem por conglomerados, esse ponto deve ser levado em consideração durante a análise, juntamente com os pesos amostrais. Dessa forma, as estimativas do Covitel devem ser corrigidas para efeito de conglomerado, além de se usar a ponderação descrita, de forma a produzir valores que representem a população das regiões e do país.

Para mais detalhes sobre tamanho, peso amostral e estimativas de precisão, ver informações no Material Suplementar (https://cadernos.ensp.fiocruz.br/static//arquivo/suppl-e00248922_8637.pdf).

### Aspectos éticos

Em acordo com a *Resolução nº 196/1996* do Conselho Nacional de Saúde (CNS), relativa à pesquisa com seres humanos, o Covitel foi submetido ao Comitê de Ética em Pesquisa da Escola Superior de Educação Física da Universidade Federal de Pelotas, sendo aprovado sob o número 5.125.635.

## Resultados metodológicos

**Figura 1:**
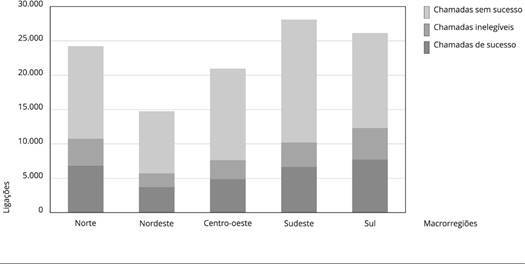
Natureza da chamada e número de ligações realizadas por macrorregião do país. *Inquérito Telefônico de Fatores de Risco para Doenças Crônicas Não Transmissíveis em Tempos de Pandemia* (Covitel), Brasil, 2022.

A coleta de dados foi executada entre os meses de janeiro e março de 2022, com divulgação de resultados preliminares em abril de 2022. Foram realizadas 114.188 ligações totais, sendo 60.371 para linhas fixas e 53.815 para móveis. Destas, 16.766 chamadas foram inelegíveis (números não existentes e/ou telefones empresariais), 29.865 chamadas de sucesso (entrevistas respondidas, agendadas, recusadas e perdas) e 67.557 chamadas sem sucesso (chamadas não atendidas, telefone desligado ou encaminhadas para secretária eletrônica). Sendo assim, representando 26,2% de taxa de sucesso, entre as 29.864 ligações válidas, 16.099 aconteceram no telefone fixo e 13.765 no celular. Quanto ao número de ligações realizadas nas diferentes regiões do país, é possível observar que a Região Nordeste apresentou o menor número de ligações realizadas; e as regiões Sul e Sudeste, os maiores. O número de ligações por região foi demonstrado na Figura 1.

Considerando resultados numéricos da aplicação das entrevistas, das ligações consideradas sucesso, houve 10.937 recusas em participar da entrevista, representando 36,6% do total. Quando estratificado por tipo de telefone, o percentual de recusa no telefone fixo foi de 34,6%; e no telefone celular, 39%. O tempo médio de duração da aplicação das entrevistas foi de 14 minutos e 53 segundos (sendo 14 minutos e 49 segundos no telefone fixo e 14 minutos e 57 segundos no telefone celular, em média).

É importante ressaltar que, tendo em vista a inferência para a população maior de 18 anos, existiu uma variação significativa dos pesos amostrais. Os pesos menores são da ordem de 1.342, chegando a um máximo de 214 mil. Os menores pesos se concentram nas regiões menos populosas (Norte e Centro-oeste), enquanto os maiores (frações amostrais menores) se concentram no Sudeste e Nordeste, entre os grupos mais jovens e de escolaridade mais alta.

## Considerações finais

Este artigo apresenta aspectos metodológicos para a execução do Covitel, inquérito telefônico realizado entre janeiro e março de 2022. Analisando questões referentes à aplicação do questionário, podem ser apontados aspectos positivos e questões que representam desafios para experiências futuras. Entre os pontos positivos, destacam-se principalmente aqueles que garantem visibilidade ao Covitel, como o uso da tecnologia de entrada eletrônica de dados. Isso evidencia grande relevância para segurança e qualidade dos dados produzidos, bem como rapidez na elaboração do banco de dados. Esse tipo de tecnologia empregada para a realização de inquéritos de vigilância tem sido utilizado em estudos similares ^7^.

Outro ponto importante em relação à metodologia do Covitel diz respeito ao método adotado para obtenção da amostra, que considera as complexidades das características demográficas da população brasileira. Portanto, o desenho amostral conseguiu compor uma amostra que permite estimar prevalências de fatores de risco com precisão razoável. Diante disso, do ponto de vista operacional, a metodologia utilizada neste estudo se mostrou positiva para a elaboração de novas pesquisas em nível nacional, além de poder ser implementada como procedimento padrão para aplicação de inquéritos telefônicos de base populacional.

A inclusão de telefones celulares foi o grande avanço metodológico do Covitel. O estudo mostrou que é possível incluir esses aparelhos, cada vez mais frequentes no cotidiano da população, em sistemas de monitoramento da saúde da população, aumentando a representatividade da amostra quando comparada com estudos de base domiciliar. Outro destaque de sucesso do inquérito realizado por telefone foi ter resultados representativos para o Brasil e para as grandes regiões. Os resultados oportunos do inquérito aqui descrito, realizado em um momento de pandemia, constituem importante entrega para o país. Eles apresentaram resultados relevantes para a construção de conhecimento sobre a influência da COVID-19 nos fatores de risco para as DCNT no Brasil, por meio da análise da situação de saúde da população em um momento ímpar, que, provavelmente, acarretará muitos desafios para as pessoas nos próximos anos.

A respeito das limitações do estudo, destacam-se três pontos principais: a possibilidade de introdução de um viés de memória relativo à abordagem do período recordatório para o momento pré-pandemia; a existência de diferenças em relação ao número de linhas telefônicas, pois sabe-se que em regiões com maior densidade populacional, geralmente capitais e regiões metropolitanas, a abrangência telefônica é maior e, dessa forma, pode introduzir um viés de seleção, que é minimizado quando se realiza a estratificação por região e pesos amostrais; e uma limitação decorrente do perfil do entrevistado, visto que, de modo geral, indivíduos que atendem o telefone fixo são pessoas que estão em casa, sem emprego e com mais idade, ou seja, estão mais suscetíveis a responder pesquisas.

A vigilância em saúde proporciona para os gestores públicos orientações para suas ações, permitindo planejamento e racionalização dos recursos, bem como propicia para a sociedade a conscientização dos problemas encontrados, que pode ocasionar melhores escolhas individuais e coletivas em prol de uma saúde melhor.
